# Optimized XGBoost Model with Small Dataset for Predicting Relative Density of Ti-6Al-4V Parts Manufactured by Selective Laser Melting

**DOI:** 10.3390/ma15155298

**Published:** 2022-08-01

**Authors:** Miao Zou, Wu-Gui Jiang, Qing-Hua Qin, Yu-Cheng Liu, Mao-Lin Li

**Affiliations:** 1 School of Aeronautical Manufacturing Engineering, Nanchang Hangkong University, Nanchang 330063, China; 2003085500156@stu.nchu.edu.cn (M.Z.); 2103085500013@stu.nchu.edu.cn (Y.-C.L.); 2 Department of Materials Science, Shenzhen MSU-BIT University, Shenzhen 518172, China; qinghua.qin@smbu.edu.cn; 3 School of Aircraft Engineering, Nanchang Hangkong University, Nanchang 330063, China; 19032@nchu.edu.cn

**Keywords:** machine learning, optimized XGBoost method, small dataset, selective laser melting, Ti-6Al-4V

## Abstract

Determining the quality of Ti-6Al-4V parts fabricated by selective laser melting (SLM) remains a challenge due to the high cost of SLM and the need for expertise in processes and materials. In order to understand the correspondence of the relative density of SLMed Ti-6Al-4V parts with process parameters, an optimized extreme gradient boosting (XGBoost) decision tree model was developed in the present paper using hyperparameter optimization with the GridsearchCV method. In particular, the effect of the size of the dataset for model training and testing on model prediction accuracy was examined. The results show that with the reduction in dataset size, the prediction accuracy of the proposed model decreases, but the overall accuracy can be maintained within a relatively high accuracy range, showing good agreement with the experimental results. Based on a small dataset, the prediction accuracy of the optimized XGBoost model was also compared with that of artificial neural network (ANN) and support vector regression (SVR) models, and it was found that the optimized XGBoost model has better evaluation indicators such as mean absolute error, root mean square error, and the coefficient of determination. In addition, the optimized XGBoost model can be easily extended to the prediction of mechanical properties of more metal materials manufactured by SLM processes.

## 1. Introduction

The Ti-6Al-4V titanium alloy is widely used in chemical, aviation, and medical fields due to its excellent mechanical properties such as high strength and high toughness, as well as its low elastic modulus and corrosion resistance [1]. However, the high cost and challenging machinability of complex components of the Ti-6Al-4V alloy restrict its applications when fabricated by traditional fabrication methods such as casting and forging [2]. Additive manufacturing (AM) is gaining increasing attention from both academia and industry due to its unique advantages over traditional isomaterial and subtractive manufacturing.

Selective laser melting (SLM) is an attractive manufacturing process for defense and other industries due to its advantages in energy utilization, absorption rate, vacuum protection, and production efficiency [1,3,4]. At present, the SLM process has been widely used in the manufacturing of Ti-6Al-4V alloys [5,6]. With the SLM process, many parameters including the laser scanning speed, laser power, hatch distance, and powder layer thickness, etc., may affect the final quality of printed products [7,8,9,10], and the ranges of these parameters are quite wide, making experiments for data collection more time-consuming and tedious. Numerous studies have reported the fabrication of high-density Ti-6Al-4V parts via SLM with different process parameters [11,12,13], and their results showed differences in the parts’ density.

Many authors have attempted to optimize SLM process parameters using various algorithms, such as direct optimization [14] and Doehlert matrix design [15]. Clearly, it is not appropriate to study process parameters independently, as the performance of the printed parts is a function of several interacting key process parameters.

In recent decades, machine learning (ML) technology has made great strides and has gone beyond the scope of computer science because it can provide a new approach to solving traditional engineering problems [16]. Dataset-based ML methods have some unique applications in the field of additive manufacturing [17,18] because of their unique advantages in data processing with high accuracy in data prediction [19].

Extreme Gradient Boosting (XGBoost), a machine learning technique, first proposed by Chen and Guestrin [20], has performed well in numerous data mining competitions due to its ability to analyze certain important parameters in the model and easily interpret the predicted output. The XGBoost-based model is a massively parallel boosted tree mode, and is currently the fastest and best boosted tree model. It is more than 10 times faster than ordinary models and has been widely used in many fields.

Some studies [21,22] have shown that XGBoost is superior to other algorithms in handling tabular datasets, such as artificial neural networks (ANN) and support vector regression (SVR), which usually require large-scale datasets in the form of pictures or videos. More recently, Duan et al. [23] used XGBoost, ANN, and SVR to predict the compressive strength of recycled aggregate concrete, and they indicated that XGBoost is better than other algorithms and the XGBoost decision tree algorithm has a very good ability to solve nonlinear regression problems.

Prominent applications of XGBoost decision trees can be found in Dong et al. [24] at the material level and Lim and Chi [25] at the structural level. Because of the recognized accuracy, XGBoost has also been used in the field of additive manufacturing in recent years. For example, Zhang et al. [26] combined XGBoost and long short-term memory (LSTM) to accurately predict the temperature in a molten pool. Through the XGBoost method, Peng et al. [27] constructed relationship mapping between the physical characteristics of defects and fatigue life of AM parts.

Conventional ML methods rely on big data [28,29], but the acquisition of big data is difficult and costly, so building ML methods for small data is particularly necessary. Yu et al. [30] developed a deep neural network (DNN) to accurately predict the tensile strength of aluminum alloys as a function of the chemical composition and process parameters with a small dataset. Normally in ML models, it is difficult to describe features with few eigenvalues, but in Yu’s work, many eigenvalues were involved [30], meaning more features were required in the manufacturing processes, and thus increasing the difficulty of obtaining the data.

For practicality and generality, this paper developed an optimized XGBoost regression tree algorithm based on supervised learning to predict the relative density of SLMed Ti-6Al-4V parts with a small dataset using hyperparameter optimization with the GridsearchCV method. Four key SLM process parameters, including the laser scanning speed, laser power, hatch distance, and powder layer thickness for regression, were used to predict the relative density of SLMed Ti-6Al-4V parts. The prediction accuracy of the proposed optimized XGBoost method was evaluated in terms of evaluation indicators such as the mean absolute error (MAE), root mean squared error (RMSE), and coefficient of determination ($R^{2}$) and was then compared with other conventional ML algorithms such as DNN [30] and SVR [31]. Determining the quality of SLMed Ti-6Al-4V parts remains a challenge due to the high cost of SLM and the need for expertise in processes and materials, which is aptly addressed by this work. The proposed optimized XGBoost model provides an alternative solution to accurately predict the relative density of SLMed Ti-6Al-4V parts with only four SLM process parameters based on a small dataset. Although this paper only discussed the application of the XGBoost model in predicting the relative density of SLMed Ti-6Al-4V parts, the optimized XGBoost model proposed in this paper can be easily extended to predict the mechanical properties of many more metallic materials fabricated by the SLM process. Therefore, the machine learning model proposed in this paper can be widely applied in the metal SLM industry.

## 2. Method

### 2.1. Experimental Dataset

The material for this study is the titanium-alloy Ti-6Al-4V ELI, which is supplied in powder form. The specific composition of Ti-6Al-4V ELI is listed in Table 1.

Choosing which experimental test dataset to use is very important to the machine learning process. The experimental dataset must be broad enough and representative of the question being studied [32]. Nguyen et al. [29] extended the original test data of 2048 sets to obtain 54,054 sets of relative density data of Ti-6Al-4V parts corresponding to the SLM process parameters. The same method was used here to obtain 54,054 sets of relative density data with the values of the laser scanning speed, laser power, hatch distance, and power layer thickness as eigenvalues via the neural network method using python programming language and the TensorFlow library. The ranges for each parameter chosen based on experience and certain references [33,34,35] are listed in Table 2.

### 2.2. XGBoost Model

**Figure 1:**
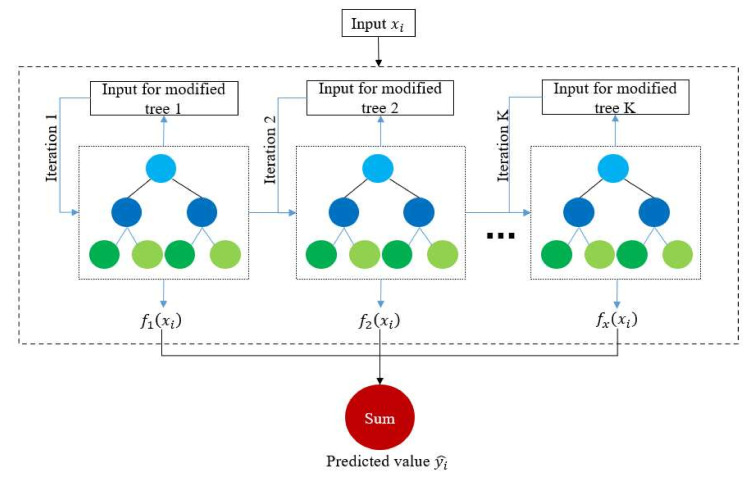
Schematic diagram of the XGBoost regression tree model.

The XGBoost algorithm utilizes many classification and regression trees (CARTs) to solve regression and classification problems. In this study, the prediction of the relative density of SLMed Ti-6Al-4V parts is a logistic regression problem. The XGBoost model is a strong regressor fused by many CART regression tree models. As shown in Figure 1, the structure of XGBoost includes multiple root nodes, internal nodes, leaf nodes, and branches. In this structure, the *i*-th parameter $x_{i}$ is input and passed to all root nodes of all CARTs to make the original decisions. Then, the internal nodes make subsequent decisions, the branch points point directly to the decision to be made, and the leaf nodes represent the prediction results of a single CART. Finally, the results of all leaf-pointing nodes are combined to obtain the prediction results of the XGBoost model [36].

As an example, in the *i*-th set $\left(x_{i},y_{i}\right)$ ($x_{i}$ is the input data with multiple features, $y_{i}$ is the real value of the trial), the XGBoost regression tree model is expressed mathematically as [20]

(1) $$\hat{y_{i}}=\alpha \overset{K}{\underset{k=1}{\sum }}f_{k}\left(x_{i}\right)$$

where $\hat{y_{i}\;}$ is the predicted value corresponding to input $x_{i}$, α is the learning rate of the individual regression tree, *K* is the total number of CARTs being used, and $f_{k}$ is the output of the *k*-th regression tree. Equation (1) shows that the predicted score $\hat{y_{i}}$ is the sum of all $f_{k}$ values.

After obtaining the prediction result, the objective function *L* was used to evaluate the quality of the obtained results, denoted as [37]

(2) $$\;L=\overset{n}{\underset{i}{\sum }}l(y_{i},\hat{y_{i}})+\overset{K}{\underset{k=1}{\sum }}\Omega \left(f_{x}\right)$$

The objective function consists of two parts: (1) The loss function *l*, measuring the loss between $\hat{y_{i}}$ and $y_{i}$ and (2) the regularization item *Ω*, determining the complexity of the regression tree structure. For a CART, *Ω* was expressed as

(3) $$\Omega \left(f\right)=\gamma T+\frac{1}{2}\lambda \overset{T}{\underset{j=1}{\sum }}\omega_{j}^{2}$$

where *T* is the total number of leaf nodes of CARTs, $\omega_{j}$ represents the predicted value of the *j*-th leaf node, and *γ* and *λ* are controlling factors employed to avoid overfitting.

To optimize the objective function and obtain the best prediction results, the XGBoost model was trained, and the optimization process was carried out step-by-step. In each step, the objective function was further reduced by generating new CART from the existing CARTs. The existing CARTs were first replaced by the constant *c*, and the equation was then subjected to second-order Taylor expansion. Based on the $L^{\left(t-1\right)}$ obtained in the previous step, the objective function $L^{\left(t\right)}$ for the *t*-th step was calculated as

(4) $$L^{\left(t\right)}=\overset{n}{\underset{i}{\sum }}[l(y_{i},{\hat{y_{i}}}^{\left(t-1\right)}+g_{i}f_{t}\left(x_{i}\right)+\frac{1}{2}h_{i}f_{t}{}^{2}\left(x_{i}\right))]+\Omega \left(f_{t}\right)+c$$

where

(5) $$\;g_{i}=\frac{\partial l\left(y_{i},{\hat{y_{i}}}^{\left(t-1\right)}\right)}{\partial {\hat{y_{i}}}^{\left(t-1\right)}}$$

(6) $$h_{i}=\frac{\partial^{2}l\left(y_{i},{\hat{y_{i}}}^{\left(t-1\right)}\right)}{\partial {\left({\hat{y_{i}}}^{\left(t-1\right)}\right)}^{2}}$$

In this study, the loss function chooses the residual standard error (RSE). Each input variable $x_{i}$ was mapped to a leaf node of a CART, so $f_{k}\left(x_{i}\right)$ was expressed as

(7) $$f_{k}\left(x_{i}\right)=\omega_{q}\left(x_{i}\right),\omega \in R^{T},q\left(x_{i}\right):R^{d}\to \left\{1,2\ldots ,T\right\}$$

where  ω is the value of this specific leaf node, $q\left(x_{i}\right)$ is the index of a specific leaf node, d is the eigenvalue of the input $x_{i}$, $R^{T}$ represents a T-dimensional vector, and $R^{d}$ represents a d-dimensional vector. The first derivative was calculated by substituting Equations (3) and (5)–(7) into Equation (4).

Letting $G_{j}=\sum_{i\in I_{j}}g_{i}$ and $\;H_{j}=\sum_{i\in I_{j}}h_{i}$, when $\;\omega_{j}=-\frac{G_{j}}{H_{j}+\lambda }$, $\;L_{min}$ was written as,

(8) $$L_{min}=\frac{1}{2}\overset{T}{\underset{j=1}{\sum }}\frac{G_{j}^{2}}{H_{j}+\lambda }+\gamma T+c$$

Therefore, the optimal value of the objective function *L* was the predicted value displayed on the leaf nodes, and in order to find the optimal structure for each CART, a greedy algorithm was used to optimize the regression tree structure [38].

### 2.3. Hyperparameter Optimization with GridsearchCV Method

According to the principle of XGBoost, certain hyperparameters, including the maximum tree depth $d_{max}\;(e.g.,\;d_{max}=3$ in Figure 1), α in Equation (1), *K* in Equation (1), γ in Equation (3), and λ in Equation (3), play a crucial role in the pros and cons of the XGBoost algorithm. The random initial values of these hyperparameters in this paper were given as follows:$\;d_{max}=3$, α=0.3, K=300, and γ=λ=0.

**Figure 2:**
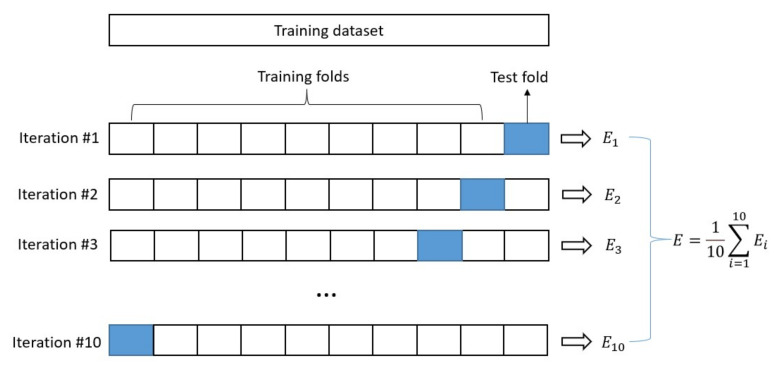
Schematic diagram of ten-fold cross-validation.

Figure 2 shows the ten-fold cross-validation used to evaluate the model. Once the values of the hyperparameter set are brought into the XGBoost model, the corresponding model is generated based on the training dataset. In this work, a ten-fold cross-validation approach was employed to improve the training accuracy by randomly dividing the training dataset into ten different subsets. The established XGBoost model was then trained and evaluated ten times, each time selecting nine subsets for training and another for evaluation. Finally, an array of ten evaluation scores (*E*) and their averages were obtained.

After the model was constructed, the accuracy of the established XGBoost model was evaluated based on the basic evaluation indicators of the three regression tasks, such as MAE, RMSE, and $R^{2}$, which were defined as

(9) $$\mathrm{MAE}\left(y,\hat{y}\right)=\frac{1}{n}\overset{n}{\underset{i=1}{\sum }}\left|y_{i}-\hat{y_{i}}\right|$$

(10) $$\mathrm{RMSE}\left(y,\hat{y}\right)=\sqrt{\frac{1}{n}\overset{n}{\underset{i=1}{\sum }}{\left(y_{i}-\hat{y_{i}}\right)}^{2}}$$

(11) $$R^{2}\left(y,\hat{y}\right)=1-\frac{\sum_{i=1}^{n}{\left(y_{i}-\hat{y_{i}}\right)}^{2}}{\sum_{i=1}^{n}{\left(y_{i}-\bar{y_{i}}\right)}^{2}}$$

where y represents the experimental values, $\hat{y}\;$ represents the values predicted by the model, and $\bar{y}$ represents the average values across the dataset.

The initial XGBoost model was established and the hyperparameters were fine-tuned using the GridsearchCV method. GridsearchCV is divided into a grid search (GridSearch) and cross-validation (CV). GridSearch is used to search for hyperparameters, that is, within the specified parameter range, and adjusts the hyperparameters in turn by step size, trains the estimator with the adjusted hyperparameters, and finds the hyperparameters with the highest accuracy in the datasets of all hyperparameters through continuous testing. The ten-fold cross-validation shown in Figure 2 was used in the CV method.

To narrow the search range and improve the efficiency of fine-tuning, a broad range of hyperparameters with different tolerances was first roughly searched on the basis of the original values, and then a fuzzy range was determined for each hyperparameter, where the tolerance ensured the accuracy of the search. Finally, all possible hyperparameter combinations were evaluated. The ranges of hyperparameters and common differences for model fine-tuning are listed in Table 3.

We combined the GridsearchCV method with the XGBoost algorithm and optimized five hyperparameters including the maximum depth $d_{max}\;$ of the tree, the learning rate α, the total number *K* of CARTs being used, the regression coefficient *γ*, and the weight coefficient *λ* by using the ten-fold cross-validation approach. Then, the test dataset was applied to the determined optimized model to evaluate its prediction accuracy and obtain the final best estimator and its hyperparameter.

## 3. Results and Discussion

### 3.1. Performance of the Optimized XGBoost Model

**Figure 3:**
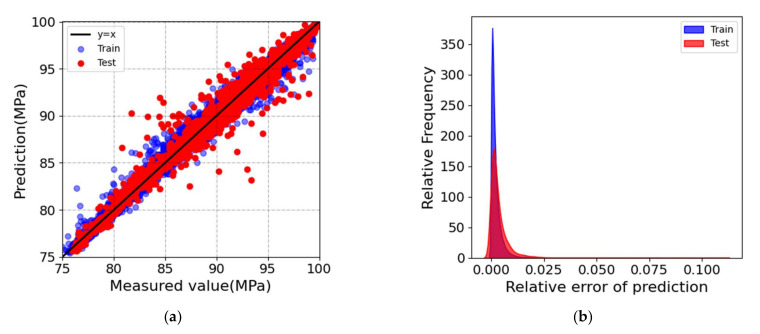
Regression analysis on the training dataset and the unseen test dataset by the trained XGBoost model. (**a**) The experimental measurement and the numerical prediction of the relative density. The solid line *y* = *x* is the identity line for reference. (**b**) Distribution plot of relative error for the training dataset and the unseen test dataset.

The best-estimated model was achieved once the best combination of hyperparameters was determined. It was then evaluated on the training and the test dataset, respectively. A dataset of 54,054 sets was generated using a Python script, which was randomly divided into training and test parts with a ratio of 8:2. Using the dataset, the depth of the XGBoost decision tree model was adjusted to 8, the learning rate was adjusted to 0.05, and the XGBoost model was trained using an ensemble of 300 regression trees. Figure 3 shows the prediction accuracy of the trained XGBoost model on the training dataset and unseen test dataset, with a total dataset size of 54,054. As shown in Figure 3a, most of the predicted points were distributed close to the straight line of *y* = *x*. Figure 3b plots the relative error distribution of the model on the training dataset and the unseen test dataset, indicating that the relative errors of the model in the training set and the test set were relatively small, and the overall prediction results were satisfactory. It could be concluded that the trained optimized XGBoost model can effectively provide an accurate relationship map between the relative density of the SLMed Ti-6Al-4V parts and the process parameters.

### 3.2. Influence of Dataset Sizes

To evaluate the generalization performance of the proposed model, the optimized XGBoost model was trained with datasets of different sizes, where all the datasets were randomly extracted from both the training part and the testing part with a ratio of 8:2. Table 4 lists the three evaluation indicators (MAE, RMSE, and *R*^2^) of the model on the unseen test set of data with different dataset sizes. Table 4 indicates that the XGBoost model proposed in this paper has superior accuracy and generalization performance in predicting Ti-6Al-4V part density when the dataset size is large. When the size of the dataset is reduced, the prediction accuracy drops but is still acceptable. For example, when the size of the test dataset is greater than 649, the coefficient of determination R2 is greater than 0.9. Even when the size of the training dataset drops to 122, the value of the coefficient for determination *R*^2^ can still reach 0.7632.

In order to verify the practicability of the model, the relative densities of SLMed Ti-6Al-4V parts were predicted by the optimized XGBoost model with different sizes of datasets proposed in this paper and were compared with the experimental measurement results of Jiang et al. [39]. The input SLM process parameters in the XGBoost model were set by the laser power of 125 W, the powder layer thickness of 20 μm, the hatch distance of 80 μm, and the laser scanning speeds of 905 mm/s, 1005 mm/s, and 1105 mm/s, respectively, which were consistent with the experimental process parameters of Jiang et al. [39]. It should be noted that the experimental data of Jiang et al. [39] were not included in our XGBoost model dataset.

**Figure 4:**
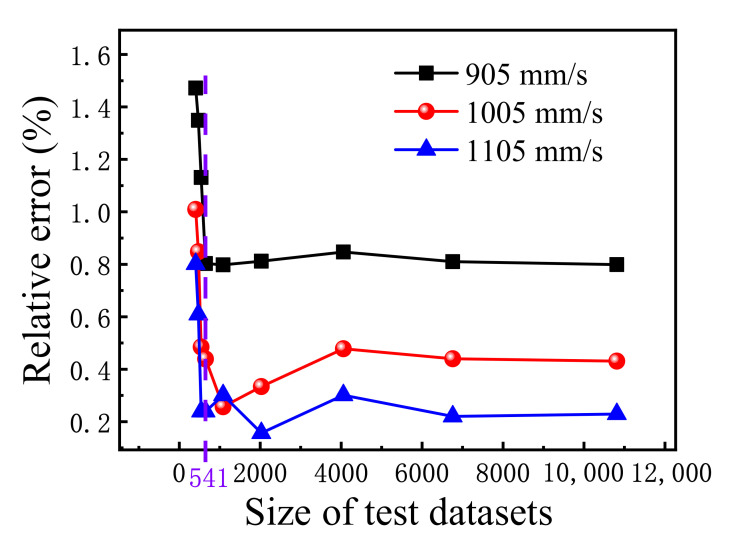
The predicted relative errors from experimental values as a function of dataset size used in the optimized XGBoost model.

Figure 4 shows the predicted relative errors from experimental values as a function of dataset size used in the optimized XGBoost model, which clearly indicates that as the dataset size decreases, when the size of the test dataset is larger than 541, the prediction accuracy changes slightly, but when the size of the test dataset is smaller than 541, the prediction accuracy drops sharply, at which it indicates that the model has lost its predictive ability. As also shown in Figure 4, the higher the scanning speed, the smaller the relative error of the model when the other three processing parameters and the dataset size are the same.

### 3.3. Comparing the Predictive Ability with That of Other ML Models under Small Dataset

In this study, a good ML model must guarantee high prediction accuracy and generalization ability under small datasets. For comparison, the SVR and DNN models were coded and trained here. We chose the test dataset of 649 sets listed in Table 4 to compare the prediction accuracy of the optimized XGBoost model with that of the SVR and DNN models.

**Figure 5:**
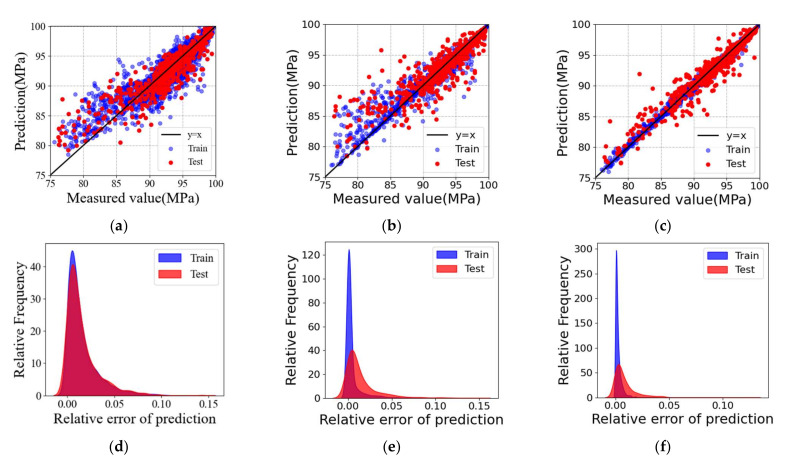
Regression analysis on the training dataset and the unseen test dataset by the trained ML models using a small dataset. The experimental measurements and the numerical predictions predicted by (**a**) DNN, (**b**) SVR, and (**c**) the present model. Distribution plot of relative error for the training dataset and the unseen test dataset evaluated in (**d**) DNN, (**e**) SVR, and (**f**) the present optimized XGBoost model.

Figure 5 shows the results of the regression analysis on the training dataset and the unseen test dataset by the trained ML models including DNN, SVR, and the present optimized XGBoost model under a small test dataset of 649 sets. The evaluation indicators, such as MAE, RMSE, and R2, of the unseen test dataset are also listed in Table 5. Both Figure 5 and Table 5 show that the optimized XGBoost model outperforms the other two models in accuracy and generality in predicting the relative density of SLMed Ti-6Al-4V parts under a small dataset. The optimized XGBoost model can effectively provide accurate correspondence between the relative density of Ti-6Al-4V parts and SLM process parameters.

## 4. Conclusions

In this study, the GridsearchCV method was used to fine-tune the hyperparameters of the XGBoost model with a small dataset to predict the correspondence between process parameters and relative densities of SLMed Ti-6Al-4V parts. The following conclusions are drawn:

(1) The trained optimized XGBoost model can effectively provide accurate correspondence between the relative density of the SLMed Ti-6Al-4V parts by SLM and the processing parameters.
(2) As the dataset size decreases, when the size of the test dataset is larger than 541, the prediction accuracy changes slightly, but when the size of the test dataset is smaller than 541, the prediction accuracy drops sharply, at which point the model has lost its predictive ability.
(3) The present optimized XGBoost model outperforms the ANN and SVR models with respect to the accuracy and generality in predicting the relative density of the SLMed Ti-6Al-4V parts under a small dataset.
(4) The optimized XGBoost model has strong practicability under a small dataset. Using this method, the SLM operators can accurately estimate the relative density of the products based on the input processing parameters before printing, without spending a great deal of experience and time.

Although the application of the XGBoost model in predicting the relative density of SLMed Ti-6Al-4V parts only was discussed here, the optimized XGBoost model proposed in this paper can be easily extended to the prediction of mechanical properties of more metal materials manufactured by SLM processes. In future work, we will further modify the model to predict the corresponding processing parameters according to the specified mechanical properties of the printed parts desired by the user.

## Figures and Tables

**Table 1:** Specific composition of Ti-6Al-4V ELI alloy powder.

Element	Al	V	Fe	C	N	O	H	Ti	Others
wt. %	5.50–6.50	3.50–4.50	0.25	0.08	0.03	0.13	0.0125	Balance	0.50

**Table 2:** SLM process parameters and their ranges used to generate data.

Process Parameters	Unit	Value
Laser scanning speed	mm/s	800, 900, 1000, 1200, 1300, 1400, 1500, 1600, 1700, 1800, 1900, 2000, 2100, 2200, 2300, 2400, 2500
Laser power	W	80, 90, 95, 100, 105, 110, 115, 120, 130, 135, 140, 145, 150, 155, 160, 165, 170, 175, 180
Hatch distance	μm	30, 35, 40, 45, 50, 55, 60, 65, 70, 75, 80, 85, 90, 95, 100
Power layer thickness	μm	20, 25, 30, 35, 40, 45, 50, 55, 60, 65, 70, 75, 80

**Table 3:** Hyperparameter ranges for model fine-tuning.

Item	Range of Values	Tolerance
$d_{max}$	1–10	1
α	0.01–0.3	0.02
*K*	100–600	50
γ	0–0.05	0.01
λ	0–1	0.1

**Table 4:** Comparison of evaluation indicators of the proposed models with different sizes of dataset on unseen test dataset.

Training Dataset (Set)	Test Dataset (Set)	MAE	RMSE	$R^{2}$
48,648	10,811	0.4768	0.6245	0.9699
27,027	6757	0.4815	0.6344	0.9696
16,216	4055	0.5194	0.7179	0.9643
8108	2028	0.6001	0.9917	0.9513
4324	1082	0.6871	1.1797	0.9428
2594	649	0.8011	1.7171	0.9184
2162	541	0.8889	2.1495	0.8930
1621	406	0.9870	2.2707	0.8840
486	122	1.5577	5.1405	0.7632

**Table 5:** Comparison of prediction results of SVR, DNN, and optimized XGBoost models on the unseen test set.

Test	SVR	DNN	Optimized XGBoost
MAE	1.3344	0.8576	0.8011
RMSE	4.8646	1.7316	1.7171
$R^{2}$	0.7687	0.7849	0.9184
